# Low-dose tenecteplase during cardiopulmonary resuscitation in massive pulmonary embolism

**DOI:** 10.1186/s12245-024-00659-5

**Published:** 2024-07-03

**Authors:** Farzin Vajifdar, Parag Badki

**Affiliations:** 1 Department of Emergency Medicine, Holy Family Multispecialty Hospital, Mumbai, India; 2 Intensive Care, Holy Family Multispecialty Hospital, Mumba, India

**Keywords:** Massive pulmonary embolism, Cardiopulmonary resuscitation, Low dose Tenecteplase

## Abstract

We report the case of an 18-year-old male who presented to the Emergency Department with sudden onset dyspnea. The patient was intubated on arrival, but suffered a cardiac arrest soon after. Point-of-care echocardiography during cardiopulmonary resuscitation revealed a grossly dilated right atrium and right ventricle, which alerted the Emergency physician to the possibility of massive pulmonary embolism leading to cardiac arrest. Due to no discernible history or risk factors in favour of pulmonary embolism, a decision was taken for thrombolysis with half dose Tenecteplase. Return of spontaneous circulation was achieved 14 min after thrombolysis, with massive pulmonary embolism subsequently being confirmed on CT Pulmonary Angiography.

## Introduction

Detection of Pulmonary Embolism (PE) in the Emergency department is a competence-based diagnosis, requiring a high index of suspicion, strong clinical acumen, and rapid focussed assessment. PE is relatively less common as compared to Acute Coronary Syndrome (ACS), so there is an inherent tendency to undermine the potential for this diagnosis in a patient presenting with chest pain or dyspnea. Additionally, PE presentations can often be vague and varied, which adds to the conundrum in diagnosis and treatment.

Unprovoked pulmonary embolism leading to cardiac arrest remains the nemesis of the Emergency Physician. Mortality in massive PE with cardiac arrest is as high as 95%, with over 70% of the cases succumbing within the first hour itself [1].

PE holds the distinction of being the only indication for thrombolysis during cardiopulmonary resuscitation (CPR). However, due to the inherent challenges faced in confirming the diagnosis during on-going CPR, and the paucity of data regarding outcomes in cases with thrombolysis during CPR, therapy with a thrombolytic agent may be delayed, or often not delivered.

Although pulmonary embolism leading to cardiac arrest is witnessed in a small percentage of in-hospital-cardiac arrests (IHCA), there is growing evidence to suggest that administration of thrombolytic therapy during cardiopulmonary resuscitation significantly improves the rates of return of spontaneous circulation as well as survival to discharge rates [2].

## Case presentation

An 18-year-old male patient was brought to the Emergency Department with complaints of sudden onset dyspnea and impending doom. On arrival, the patient was found to be profusely diaphoretic with a Heart rate of 148 beats per minute (sinus tachycardia), saturation of 60% on room air, Respiratory rate of 44 per minute with significant respiratory distress and equal chest rise on both sides. Breath sounds were equal bilaterally, with no adventitious sounds and chest was clear. Heart sounds were normal, with no murmurs.

Patient was combative, agitated, with a GCS of 11 (E4V2M5), pupils were bilateral 3 mm, briskly reactive. Random blood sugar was 165 mg/dl. Unable to record the Blood pressure on arrival due to the combative nature of the patient, however subsequently the Systolic blood pressure was found to be 60 mm Hg. Patient was profusely diaphoretic with cold, clammy extremities, and acrocyanosis was noted over the lips.

A decision was taken to intubate the patient in view of respiratory failure with impending arrest. A rapid sequence induction was done, and patient was intubated and mechanically ventilated. Shortly after intubation, there was loss of central pulses, and CPR was initiated as per ACLS protocol. Initial rhythm was Pulseless Electrical Activity (PEA), which remained persistent through the CPR.

A point-of-care ultrasonography (POCUS) performed during CPR (in the intervals for pulse checks) showed a plethoric inferior vena cava (IVC) and massively dilated right atrium and right ventricle on the apical view, while a short axis view showed the right ventricle indenting the left ventricle (D-cup sign). A presumptive diagnosis of massive pulmonary embolism was made during CPR, and a decision was taken for thrombolysis of the patient after taking the family into confidence.

Owing to no discernible history or risk factors suggestive of pulmonary embolism, it was decided to use only 20 mg of Tenecteplase. Intravenous Tenecteplase was administered with ongoing CPR (about 8 min into CPR). Return of spontaneous circulation (ROSC) was achieved 14 min after thrombolysis, with a total duration of 22 min of CPR. Targeted temperature control was initiated and patient was shifted to ICU, with the CT Pulmonary Angiography deferred until stabilization of the patient.

Subsequently a CT Pulmonary Angiography (CT-PA) was done, which showed a large filling defect in the left main pulmonary artery and bilateral descending pulmonary arteries, thereby confirming the diagnosis of massive pulmonary embolism leading to cardiac arrest. Mechanical thrombo-suction was further done for the patient after which he had an insidious improvement.

## Differential diagnosis

Drug overdose / Toxidrome

Septic Shock with nidus as Lower respiratory tract infection

Adult Respiratory Distress Syndrome

Myocarditis

## Treatment and outcome

The patient had a protracted ICU stay with multi-organ involvement (including acute renal injury requiring dialysis, an ischaemic stroke, intra-abdominal bleed, hemothorax, and a difficult weaning requiring tracheostomy). It is worth noting that the bleeding complications were delayed, and arose much later in the course of hospital stay, and therefore could also be attributed to the concurrent use of anticoagulants. However, with a dedicated team and excellent inter-disciplinary approach, he went on to make a full recovery (barring a mild residual left upper arm paresis in the non-dominant arm secondary to the ischaemic stroke).

Blood work up for a pro-thrombotic state was negative, bilateral lower limb venous doppler was negative for deep vein thrombosis, and there were no precipitating or predisposing factors for pulmonary embolism (example recent travel history or immobilization) in this case. It was therefore labelled as a case of unprovoked, massive pulmonary embolism leading to cardiac arrest.

## Discussion

Confirming a clinical suspicion of pulmonary embolism in a critically ill patient in the Emergency Department (ED) poses a unique challenge as these patients are often too unstable to be mobilized for a CT pulmonary angiography (CT-PA), which is the gold standard for diagnosis. In such cases, a rapid, focused, bed-side point-of-care ultrasonography (POCUS) can greatly aid in the diagnosis and decision-making process [3].

Cardiac arrest following pulmonary embolism carries a mortality of 70% in the first hour itself, while the overall mortality may reach 95% [1]. Confirmed or suspected pulmonary embolism as the cause of cardiac arrest is an indication to administer thrombolytic therapy during cardiopulmonary resuscitation (CPR) as per the American Heart Association (AHA) recommendations for Advanced Cardiac Life Support (ACLS). There is increasing evidence to support the use of Alteplase and Tenecteplase for thrombolysis in massive pulmonary embolism, especially when leading to cardiac arrest [2, 4].

A systematic literature review showed only one other case of Thrombolysis with Low dose (20 mg) Tenecteplase during CPR [5] (a 29-year-old male who suffered a massive PE requiring CPR while undergoing a complex tibial plateau fracture repair). In this case, 20 mg Tenecteplase was administered after 70 min of CPR, and ROSC was achieved after 2 min of CPR.

However, there have been other case reports citing full dose Tenecteplase, as well as Alteplase for thrombolysis during CPR in case of massive PE causing cardiac arrest, and subsequently leading to favourable outcomes [6–9]. Of these, bleeding as a major complication was reported in only one case of a 34-year-old woman undergoing an emergency Caesarean section, who suffered from massive uterine bleeding after thrombolysis with Tenecteplase for massive PE during CPR [9].

Use of Low-dose Tenecteplase had a favourable outcome in our patient despite the protracted hospital stay. Considering the extremely poor survival in massive PE with cardiac arrest, thrombolysis during CPR may give the best chance at survival in the acute phase of resuscitation, as well as discharge.

It is also worth noting that in our case, a favourable end-outcome was achieved with half dose Tenecteplase. This may therefore be considered in patients with haematological disorders or other contraindications to full dose Tenecteplase. However, it is essential to note that there may be delayed bleeding complications due to concurrent use of anticoagulants. More research in this area is warranted.

There were 3 things that added to the novelty of this case:

- ROSC achieved with Low dose Tenecteplase.
- Late bleeding complications observed despite Low dose Tenecteplase.
- Unprovoked, massive PE leading to cardiac arrest, which made the diagnosis a challenge, and the decision for thrombolysis up for debate.

## Conclusion

Although pulmonary embolism as the cause of cardiac arrest is seen in a small number of in-hospital-cardiac arrests (IHCA), thrombolysis during CPR should be strongly considered when massive pulmonary embolism is known or strongly suspected as the cause of cardiac arrest. There is growing evidence to suggest that administration of thrombolytic therapy during CPR can significantly improve the rate of ROSC and survival to discharge rates for such patients [2, 10].

## Data Availability

No datasets were generated or analysed during the current study.

## Abbreviations

ACS: Acute coronary syndrome
CPR: Cardiopulmonary resuscitation
CT-PA: Computed Tomography pulmonary angiography
ED: Emergency Department
GCS: Glasglow Coma Scale
ICU: Intensive care unit
IHCA: In-hospital cardiac arrest
PE: Pulmonary embolism
PEA: Pulseless electrical activity
POCUS: Point-of-care ultrasonography
ROSC: Return of spontaneous circulation

## References

[1] Laher AE, Richards G, Indian Heart J. 2018 Sep-Oct;70(5):731–5. 10.1016/j.ihj.2018.01.014. Epub 2018 Jan 8. PMID: 30392514; PMCID: PMC6204441. 10.1016/j.ihj.2018.01.014 PMC6204441 30392514

[2] Henriksson CE, Frithiofsson J, Bruchfeld S, Bendz E. Maria Bruzelius, Therese Djärv, In-hospital cardiac arrest due to pulmonary embolism – Treatment and outcomes in a Swedish cohort study, Resuscitation Plus, Volume 8, 2021, 100178, ISSN 2666–5204, 10.1016/j.resplu.2021.100178. 10.1016/j.resplu.2021.100178 PMC8571515 34766067

[3] Borloz MP, Frohna WJ, Phillips CA, Antonis MS. Emergency department focused bedside echocardiography in massive pulmonary embolism. J Emerg Med. 2011;41(6):658 – 60. 10.1016/j.jemermed.2011.05.044. Epub 2011 Aug 4. PMID: 21820258. 10.1016/j.jemermed.2011.05.044 21820258

[4] Ucar EY (2019). Update on thrombolytic therapy in Acute Pulmonary Thromboembolism. Eurasian J Med.

[5] Hefer DV, Munir A, Khouli H. Low-dose tenecteplase during cardiopulmonary resuscitation due to massive pulmonary embolism: a case report and review of previously reported cases. Blood Coagul Fibrinolysis. 2007;18(7):691-4. 10.1097/MBC.0b013e3282a167a7. PMID: 17890959. 10.1097/MBC.0b013e3282a167a7 17890959

[6] Yu Y, Zhai Z, Yang Y, Xie W, Wang C (2017). Successful thrombolytic therapy of post-operative massive pulmonary embolism after ultralong cardiopulmonary resuscitation: a case report and review of literature. Clin Respir J.

[7] Greco F, Misuraca G, Serafini O, Guzzo D, Plastina F (2001). Terapia Trombolitica durante rianimazione cardiopolmonare per embolia polmonare acuta massiva. Descrizione Di Un caso clinico [Thrombolytic therapy during cardiopulmonary resuscitation for acute massive pulmonary embolism. A case report]. Minerva Cardioangiol.

[8] Abdulla W, Netter U (2005). Case report. Successful use of tenecteplase in massive pulmonary embolism with cardiopulmonary resuscitation immediately following tracheostomy. Acta Anaesthesiol Belg.

[9] Wenk M, Pöpping DM, Hillyard S, Albers H, Möllmann M. Intraoperative thrombolysis in a patient with cardiopulmonary arrest undergoing caesarean delivery. Anaesth Intensive Care. 2011;39(4):671-4. 10.1177/0310057X1103900422. PMID: 21823388. 10.1177/0310057X1103900422 21823388

[10] Summers K, Schultheis J, Raiff D, Dahhan T (2019). Evaluation of rescue thrombolysis in Cardiac arrest secondary to suspected or confirmed pulmonary embolism. Ann Pharmacother.

